# Negative pressure ventilation protects the brain

**DOI:** 10.1186/s13054-022-04150-6

**Published:** 2022-10-31

**Authors:** C. M. van Rijn, J. van Egmond, D. Howard, M. G. Coulthard, P. Perella, J. H. M. Roberts, D. McKeown

**Affiliations:** 1 Donders Institute for Brain, Cognition and Behaviour, Donders Centre for Cognition, Radboud University, Thomas van Aquinostraat 4, 6525 GD Nijmegen, The Netherlands; 2 Charing Cross Hospital, London, UK; 3 Translational and Clinical Research Institute, Newcastle University, Newcastle upon Tyne, UK; 4 The Royal National Ear, Nose and Throat and Eastman Dental Hospitals, UCLH, London, UK; 5 Exovent Charity, London, UK

Dear Editor,

With great interest we have read the recent paper in the journal 'Critical Care,' titled: ‘The future of intensive care: delirium should no longer be an issue,’ by Kotfis et al. [1]. Kotfis et al. state that the major factor to prevent delirium on an intensive care unit (ICU) is an awake, non-sedated patient [1]. However, the standard mechanical ventilation support on the ICU is through positive pressure ventilation (PPV), and in the cases that this ventilation support requires intubation of the patient, sedatives are frequently administered. Sedation is a major risk factor to develop delirium [2, 3]. Delirium is harmful for the brain as it is associated with long-term cognitive impairment [3].

Kotfis et al. recommend that new technologies should be implemented for delirium prevention [1]. In response to that suggestion, we would like to draw the attention of the ICU community to the reintroduction of negative pressure respiratory support [4, 5]. Patients receiving ventilation support by negative pressure ventilation (NPV) do not require intubation, so the need for sedation is greatly reduced. Therefore, NPV will avoid one of the major risk factors for delirium: sedatives. Moreover, since patients remain conscious during negative pressure ventilation support, the medical staff and family can continue to communicate with the patients. This diminishes the risk of developing a post-intensive care syndrome (PICS), which includes not only cognitive decline but also psychiatric symptoms like depression and post-traumatic stress disorder (PTSD) [1, 2].

By avoiding the use of sedatives negative pressure respiratory support protects the brain. NPV may prove to be a worthy addition to the current range of respiratory support strategies [4, 5].

## Data Availability

Not applicable.

## Abbreviations

ICU: Intensive care unit
NPV: Negative pressure ventilation
PICS: Post-intensive care syndrome
PPV: Positive pressure ventilation
PTSD: Post-traumatic stress disorder
