# Supplementary material for: Negative pressure ventilation protects the brain
Source: Crit Care. 2022 Oct 31;26:334. doi: 10.1186/s13054-022-04150-6 (PMC9624026; doi:10.1186/s13054-022-04150-6)
Supplement: Related texts

# Author’s reply to Correspondence “Negative pressure ventilation protects the brain”

**Authors:** Katarzyna Kotfis, Irene van Diem-Zaal, Shawniqua Williams Roberson, Marek Sietnicki, Mark van den Boogaard, Yahya Shehabi, E. Wesley Ely

**Affiliations:** 6 grid.107950.a 0000 0001 1411 4349 Department of Anesthesiology, Intensive Therapy and Acute Intoxications, Pomeranian Medical University, Szczecin, Poland; 7 grid.10417.33 0000 0004 0444 9382 Department of Intensive Care, Radboud Institute for Health Sciences, Radboud University Medical Center, Nijmegen, The Netherlands; 8 grid.7692.a 0000000090126352 Department of Intensive Care Medicine, University Medical Center Utrecht, Utrecht, The Netherlands; 9 Critical Illness, Brain Dysfunction, and Survivorship (CIBS) Center, Center for Health Services Research, Nashville, TN USA; 10 grid.412807.8 0000 0004 1936 9916 Department of Neurology, Vanderbilt University Medical Center, Nashville, TN USA; 11 grid.152326.1 0000 0001 2264 7217 Department of Biomedical Engineering, Vanderbilt University, Nashville, TN USA; 12 grid.411391.f 0000 0001 0659 0011 Department of Architecture, West Pomeranian University of Technology in Szczecin, Szczecin, Poland; 13 grid.1002.3 0000 0004 1936 7857 Monash Health School of Clinical Sciences, Monash University, Melbourne, VIC Australia; 14 grid.1005.4 0000 0004 4902 0432 School of Clinical Medicine, University of New South Wales, Sydney, NSW Australia; 15 grid.412807.8 0000 0004 1936 9916 Division of Allergy, Department of Medicine, Pulmonary, and Critical Care Medicine, Vanderbilt University Medical Center, Nashville, TN USA; 16 Geriatric Research, Education and Clinical Center (GRECC) Service, Nashville Veterans Affairs Medical Center, Tennessee Valley Healthcare System, Nashville, TN USA

We would like to thank van Rijn and colleagues for taking time to read our Viewpoint recently published in Critical Care [1] and raising attention of the possible benefits of continuous external negative-pressure ventilation (CENPV) in the context of delirium. We agree that excessive sedation is one of the major factors contributing to the development of delirium in the intensive care unit (ICU) as is intubation and continuous positive-pressure ventilation (CPPV). The COVID-19 pandemic, with the initial ventilator shortage, has been an overwhelming global challenge, but it also brought our attention to technology advances in the field of mechanical ventilation and towards the use of modalities that do not require tracheal intubation.

Experimental data have shown that continuous external negative-pressure ventilation simulates natural lung movements, reduces the incidence of barotrauma and adverse cardiovascular events [6]. Negative-pressure ventilation provides more physiological inflation of the lung volume during both inspiration and expiration and may result in better oxygenation and less lung injury as compared with CPPV [6]. According to Raymondos and colleagues in patients with acute respiratory distress syndrome (ARDS) CENPV with tank respirator leads to improved gas exchange at lower transpulmonary, airway and intraabdominal pressures [7].

However, the effects of CENPV on the brain and its association with delirium remain unknown. Palmer et al. reported a decrease in cerebral blood volume and a decrease in both oxygenated and deoxygenated hemoglobin during CENPV in newborns, suggesting increased venous drainage from the cerebral circulation during negative pressure ventilation [8].

Nevertheless, to decrease the incidence of delirium many overlapping factors must be considered. The development of delirium in critically ill patients is multifactorial and does not entirely rely on sedation practices, but rather on the way we manage critically ill patients in general [9]. With CENPV a range of possible factors contributing to delirium exist that include limited access to patients for nursing procedures, possible sensory deprivation and muscle wasting through prolonged immobilization, limited physiotherapy, and poor sleep hygiene. Moreover, the nursing workload is likely to be increased. Despite unquestionable advantages, CENPV due to its robust size and technical requirements that causes limited mobility may be another factor predisposing the patients to delirium. Therefore, further studies are necessary in this field. The mainstay of delirium management is humanitarian care through good sedation practices, excellent pain relief, respiratory support personalized to suit patients’ individual needs, and optimal non-pharmacological support in healing environment that minimizes stressors [1, 9].
